# Myofibroblastoma of the Female Breast with Admixed but Distinct Foci of Spindle Cell Lipoma: A Case Report

**DOI:** 10.1155/2013/738014

**Published:** 2013-12-29

**Authors:** Hazem A. H. Ibrahim, Sami Shousha

**Affiliations:** ^1^Department of Histopathology, Laboratory Block, Charing Cross Hospital Campus, Imperial College Healthcare NHS Trust and Imperial College, Fulham Palace Road, London W6 8RF, UK; ^2^Department of Histopathology, Faculty of Medicine, Mansoura University, Mansoura 35516, Egypt

## Abstract

Mammary myofibroblastoma (MFB) is a rare benign spindle neoplasm that affects both sexes with a male predominance. It can exhibit a wide range of histological patterns. We report a case of epithelioid/spindle MFB of the female breast with admixed, but distinct, foci of spindle cell lipoma. Whilst all the spindle cells within the tumour expressed CD34, AR, ER, BCL2, and CD10, only those within the myofibroblastoma expressed desmin and only those within the lipomatous areas expressed S100. This finding, to our knowledge, is a novel one that has not been reported before.

## 1. Introduction

Mammary myofibroblastoma (MFB) is a rare benign spindle neoplasm that affects both sexes with a male predominance. It can exhibit a wide range of histological patterns including (1) collagenized/fibrous, (2) cellular, (3) lipomatous, (4) infiltrative, (5) myxoid, (6) epithelioid, and (7) deciduoid-like variant [1, 2].

There is a case of myofibroblastoma of the male breast with pleomorphic lipoma-like areas [3]. There have been some case reports of breast tumours with morphologic features similar to spindle cell lipoma of soft tissue, solitary fibrous tumour, and myofibroblastoma of the breast and were classified as benign spindle cell tumours of the breast. Magro et al. [4] reported in a series of 13 cases of benign spindle stromal tumour of the breast 2 cases of solitary fibrous tumour and spindle cell lipoma-like (SCL-like) tumour, 9 cases of Myofibroblastomas, and 2 cases of mixed benign spindle stromal tumour (BSST). The latter included a case of myofibroblastoma with focal solitary fibrous tumour and pleomorphic/SCL-like areas and SFT with focal MFB-like component. It has been suggested that these nonmyofibroblastomatous spindle cell lesions likely represent the precursor of the evolving myofibroblastoma [4, 5].

We report a case of epithelioid/spindle MFB of the female breast with admixed, but distinct, foci of spindle cell lipoma. This finding, to our knowledge, is a novel one that has not been reported before.

## 2. Case Report 

An elderly female patient was admitted to Charing Cross Hospital for breast screening. Mammography and ultrasound identified a small nodule in her left breast suggestive of a benign lesion, possibly a fibroadenoma. This was then sampled with an US guided core biopsy. The histology showed fibroadenomatoid change in one core. The other core demonstrated groups of ovoid/epithelioid and fascicles of bland spindle cells separated by bundles of hyalinised collagen. These lesional cells were positive for desmin and negative for AE1/3, S100, and SMA. There was no evidence of DCIS or malignancy. An excision biopsy was advised to enable further characterization of the lesion.

The wide local excision specimen on slicing revealed a 12 mm well-defined firm grey nodule. Sections were stained for H&E and a number of antibodies including AE1/3, CK5, S100, SMA, Desmin, CD34, BCL2, ER, AR, and CD10.

## 3. Results 

**Figure 1:**
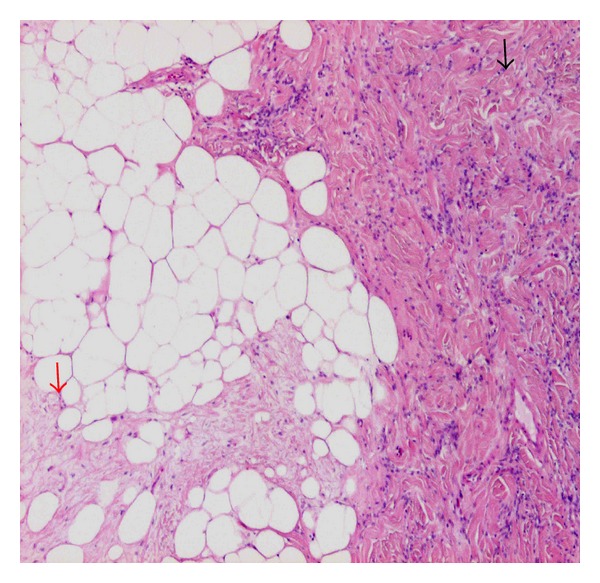
Mammary myofibroblastoma (MFB) with admixed but distinct spindle cell lipoma component. MFB component (black arrow) shows clusters of bland ovoid/spindle or epithelioid cells set in a dense fibrous stroma and spindle cell lipoma component (red arrow) (H&E (×200)).

**Figure 2:**
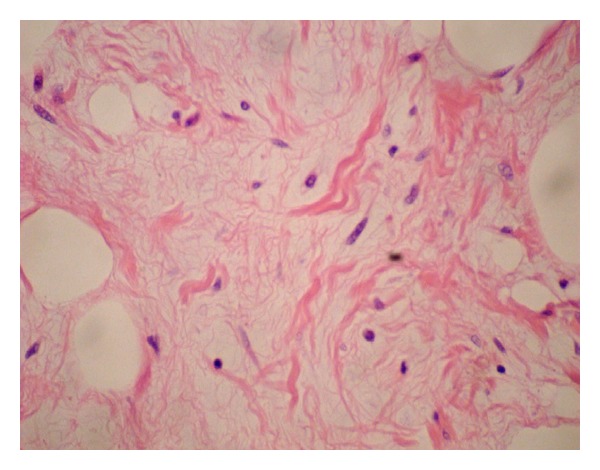
Spindle cell lipoma component shows mature fat cells with intervening bland spindle cells, set within loose/myxoid stroma containing ropey collagen, H&E, 600x.

Microscopic examination of the H&E sections demonstrated a well circumscribed, nonencapsulated lesion composed of bland slender, spindle, and epithelioid cells with moderate amount of cytoplasm arranged haphazardly in fascicles and in clusters separated by thick hyalinised bundles of collagen. These features were considered to be consistent with a myofibroblastoma (Figure 1). Occasional entrapped benign glandular structures were also seen. In addition, the lesion contained three distinct lobules of adipose tissue that formed about 10% of the lesion. Each lobule was composed of mature fat cells with intervening bland spindle cells, set within loose/myxoid stroma containing ropey collagen and occasional mast cells (Figures 1 and 2). These features were considered to be consistent with a spindle cell lipoma. There was no necrosis and no mitoses were identified within the whole lesion. Background breast tissue showed fibrocytic change.

**Figure 3:**
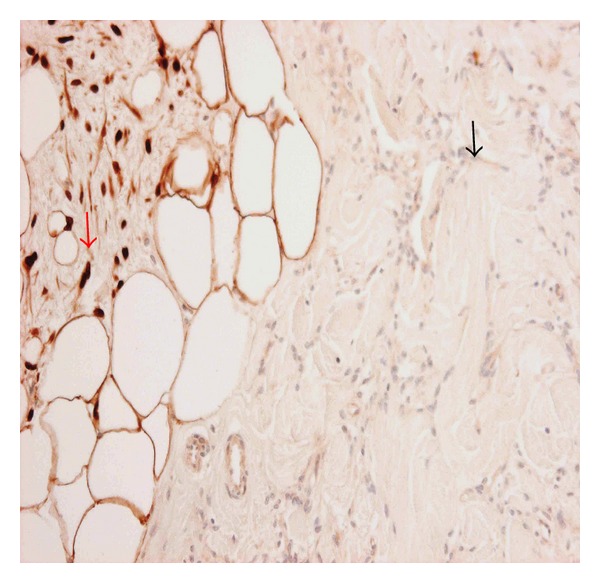
Mammary myofibroblastoma (MFB) with admixed but distinct spindle cell lipoma component. S-100 decorates spindle cells of spindle cell lipoma component (red arrow) but not MFB component (black arrow), immunoperoxidase, 400x.

**Figure 4:**
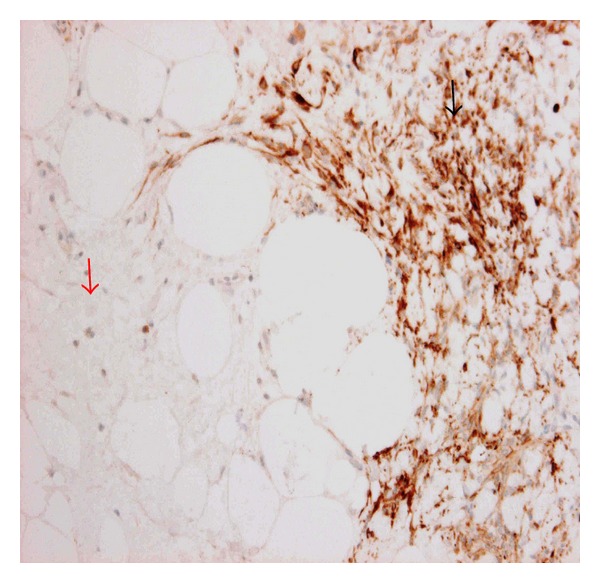
Mammary myofibroblastoma (MFB) with admixed but distinct spindle cell lipoma component. Desmin stains spindle cell of myofibroblastoma component (black arrow) but not spindle cell lipoma component (red arrow), immunoperoxidase, 400x.

**Figure 5:**
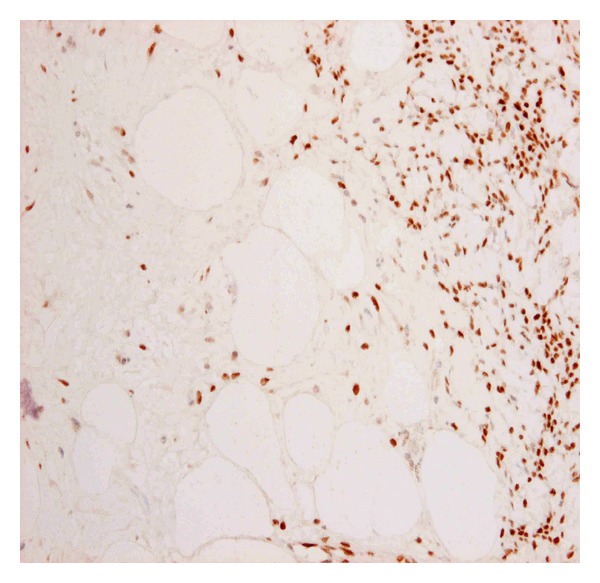
Mammary myofibroblastoma (MFB) with admixed but distinct spindle cell lipoma component. Spindle cells of both components express androgen receptor (stronger in the MFB component), immunoperoxidase, 400x.

Immunoperoxidase staining showed that the main tumour cells expressed desmin and lacked S100 expression (Figures 3 and 4). In contrast, the fat cells and locally mingled spindle cells were negative for desmin and strongly positive for S100 (Figures 3 and 4). The main tumour spindle-shaped cells as well as those entrapped within the fat lobules were positive for CD34, BCL2, ER, AR (Figure 5), and CD10 (weak). All spindle cells lacked expression of AE1/AE3, CK5, and SMA, although AE1/AE3 highlighted the entrapped benign ducts (images not shown). This immunoprofile was interpreted as being consistent with a benign mixed myofibroblastoma and spindle cell lipoma.

## 4. Discussion 

Myofibroblastoma of the breast is a rare lesion that can show a wide variety of different morphologic patterns some of which can be similar to other spindle-cell benign tumours of the breast, like solitary fibrous tumour (SFT) and spindle cell lipoma (SCL). The recognition of these patterns is vital to avoid erroneous confusion with malignant lesions [1, 4, 5]. We report a novel case of a mixed myofibroblastoma with occasional intermingled benign glands and spindle cell lipoma. This differs from the previously characterised lipomatous myofibroblastoma because it has a distinct spindle cell component within the lipomatous areas that also have a specific immunoprofile. It also differs from the published case report of myofibroblastoma arising from hamartoma where a typical hamartomatous component (comprised nearly 50% of the whole lesion) was present [6]. The typical morphology and immunoprofile in our case excluded the diagnosis of Hamartoma of the breast which was considered in the differential diagnosis. However, the occasionally dispersed benign glands within the MFB are a rare unpublished finding [6]. In addition, they did not show a lobular arrangement and were not accompanied by mature fat cells as we would see in Hamartoma. Whilst all the spindle cells within the tumour expressed CD34, AR, ER, BCL2, and CD10, only those within the myofibroblastoma expressed desmin and only those within the lipomatous areas expressed S100. Maggiani et al. [7] examined a group of mesenchymal tumours, including myofibroblastoma, spindle cell lipoma, and hybrid tumours, and identified mesenchymal CD34+ stem cells that were capable of both fibroblastic differentiation (alone or in combination with lipocytic differentiation) and myofibroblastic differentiation. The pathway taken by these cells dictated the histology of the final tumour. Furthermore, spindle cell and pleomorphic lipomas share characteristic losses of 13 q and/or 16 q loci [8], a feature that has also been described within mammary-type myofibroblastoma [9]. The difference in desmin expression highlights the two distinct pathways of differentiation that these precursor stem cells take, one of which follows a fibroblastic/lipocytic lineage, losing desmin expression, to form a spindle cell lipoma, and the other follows a myofibroblastic lineage, retaining desmin expression, to produce a myofibroblastoma [9]. Magro et al. [4] provided a unifying histogenetic concept for all benign spindle stromal tumours (BSST) of the breast and suggested the term BSST of the breast for the following entities: spindle cell lipoma-like tumour, solitary fibrous tumour, and myofibroblastoma of the breast.

## 5. Conclusion

Herein, we report a rare case of epithelioid/spindle MFB of the female breast with admixed, but distinct, foci of spindle cell lipoma. This finding, to the best of our knowledge, has not been reported before. Our case reinforces the close relationship between spindle cell lipoma and myofibroblastoma and supports the concept suggested by Magro et al., [4] which is that BSST are morphological and immunophenotypical variants within a continuous biological spectrum.
